# An intelligent monitoring system of diseases and pests on rice canopy

**DOI:** 10.3389/fpls.2022.972286

**Published:** 2022-08-11

**Authors:** Suxuan Li, Zelin Feng, Baojun Yang, Hang Li, Fubing Liao, Yufan Gao, Shuhua Liu, Jian Tang, Qing Yao

**Affiliations:** ^1^School of Computer Science and Technology, Zhejiang Sci-Tech University, Hangzhou, China; ^2^State Key Laboratory of Rice Biology, China National Rice Research Institute, Hangzhou, China

**Keywords:** disease and pest lesions, rice canopy, intelligent monitoring system, network camera, deep learning, YOLOv4 detection model

## Abstract

Accurate and timely surveys of rice diseases and pests are important to control them and prevent the reduction of rice yields. The current manual survey method of rice diseases and pests is time-consuming, laborious, highly subjective and difficult to trace historical data. To address these issues, we developed an intelligent monitoring system for detecting and identifying the disease and pest lesions on the rice canopy. The system mainly includes a network camera, an intelligent detection model of diseases and pests on rice canopy, a web client and a server. Each camera of the system can collect rice images in about 310 m^2^ of paddy fields. An improved model YOLO-Diseases and Pests Detection (YOLO-DPD) was proposed to detect three lesions of *Cnaphalocrocis medinalis, Chilo suppressalis,* and *Ustilaginoidea virens* on rice canopy. The residual feature augmentation method was used to narrow the semantic gap between different scale features of rice disease and pest images. The convolution block attention module was added into the backbone network to enhance the regional disease and pest features for suppressing the background noises. Our experiments demonstrated that the improved model YOLO-DPD could detect three species of disease and pest lesions on rice canopy at different image scales with an average precision of 92.24, 87.35 and 90.74%, respectively, and a mean average precision of 90.11%. Compared to RetinaNet, Faster R-CNN and Yolov4 models, the mean average precision of YOLO-DPD increased by 18.20, 6.98, 6.10%, respectively. The average detection time of each image is 47 ms. Our system has the advantages of unattended operation, high detection precision, objective results, and data traceability.

## Introduction

Rice is one of the most important food crops in the world. Every year, rice diseases and pests cause huge losses of yields which are a threat to global food security (Ali et al., 2019; Jiang et al., 2020; Liu et al., 2020). Therefore, real-time and accurate monitoring and forecasting of diseases and pests are very important to effectively control them and prevented yield reduction. At present, the monitoring method of diseases and pests on rice canopy in a small-scale region still relies on the manual survey in paddy fields. Agricultural technicians investigate the disease and pest lesions on rice leaves and stems in paddy fields, then determine the damage grade through visual measurement (Sethy et al., 2020). So the manual survey method has high labor intensity and low work efficiency. Its accuracy depends on the surveyors’ experiences. It is difficult to meet the real-time monitoring of diseases and pests on rice canopy.

In recent years, with the development of unmanned aerial vehicles (UAV), low-altitude remote sensing technology has been applied in monitoring agricultural diseases and pests because of UAV’s high automation and flexibility. The UAVs carry multispectral cameras or visible light cameras to collect rice images. Some image processing methods and models were proposed to identify and estimate diseases and pests (Huang et al., 2018; Zhang et al., 2018, 2019; Yang et al., 2019; Abd El-Ghany et al., 2020; Aboutalebi et al., 2020; Gao et al., 2020; Morsy, 2020; Lin et al., 2020a). However, the rotating rotors of UAVs easily cause downdrafts, which sway the rice leaves. The camera in the UAV can hardly collect high-quality images of disease and pest lesions on the rice canopy. In addition, using UAVs to collect lesion images still needs professional UAV pilots and high costs, which affects the promotion and application of UAVs in monitoring rice diseases and pests. So more efficient and convenient monitoring methods should be studied.

With the rapid development of artificial intelligence, many deep learning models have been widely used in crop plant growth (Yasrab et al., 2021), health analysis (Joshi et al., 2021) and yield estimation (Khaki et al., 2021) and so on in recent years. Especially, the YOLO model greatly improves the detection speed while satisfying higher precision and can realize real-time target detection. Some improved YOLO models were used to automatically detect agricultural disease and pest lesions and achieved good detection results. Tian et al. (2019) proposed an improved YOLOv3 model for real-timely detecting anthracnose lesions on apple surfaces in orchards. The model achieved an accuracy of 95.57%. Liu and Wang (2020a) proposed an early recognition method of tomato leaf spots based on MobileNetv2-YOLOv3 model to achieve a good balance between the accuracy and real-time detection of tomato gray leaf spot. Liu and Wang (2020b) used the image pyramid method to optimize the feature layer of the YOLOv3 model, which had a good effect on detecting tomato diseases and pests in natural environment.

The automatic detection and identification methods of rice disease and pest lesions have made big progress. Lu et al. (2017) developed a small hand-crafted CNN network model to classify an image into one of 10 common rice diseases, but could not detect the location and quantity of rice diseases and pests. Zhou et al. (2019) proposed a method of fusing Faster R-CNN with FCM-Km for detecting rice diseases which solved various problems of rice disease images, such as noise, blurred image edges, complex background interference and low detection accuracy. Li et al. (2020) proposed a video detection architecture based on deep learning and custom backbone, which was used for detecting rice diseases and pests in videos. Bari et al. (2021) added the RPN structure into the Faster R-CNN algorithm to accurately locate the target position for generating candidate regions, which had a good detection effect on three diseases on rice leaves of one plant. Daniya and Vigneshwari (2021) proposed a deep neural network model to detect rice diseases. First, the background noise of the rice disease images was removed. Then the SegNet network was used for segmentation. CNN, texture and statistical features were extracted for detection. However, these methods have not been widely used in detecting and identifying diseases and pests in paddy fields. There are mainly the following problems: (1) Some images were collected in lab conditions rather than in paddy fields. In fact, the lesions of disease and pest appear different image features under complex field environments. The models obtained in the lab could not achieve a good identification effect under fields. (2) Some studies only identified the lesions of disease and pest on one leaf. These results are unable to directly apply to identifying multi-lesions from many rice plants in one image. (3) Most image acquisition devices still needed the manual operation. In order to solve the above problems and further improve the monitoring intelligence of pests and diseases on rice canopy in paddy fields, we developed an intelligent monitoring system for detecting and identifying lesions of disease and pest on rice canopy.

## Materials and methods

### Intelligent monitoring system architecture

**Figure 1:**
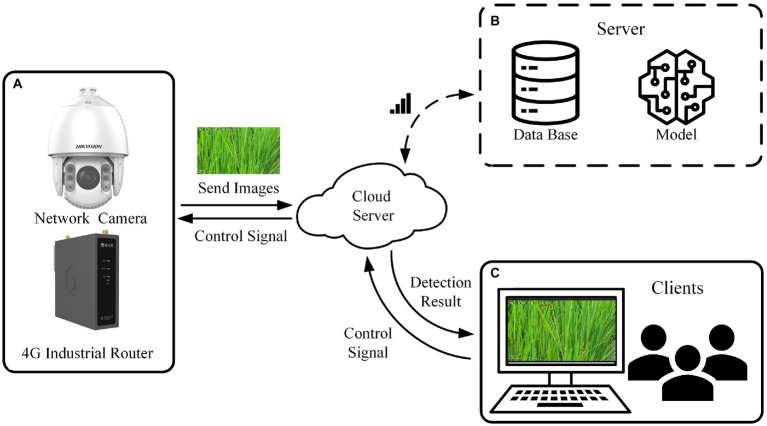
An intelligent monitoring system for rice diseases and pests.

The intelligent monitoring system of rice diseases and pests consists of an image acquisition device (Figure 1A), a cloud server (Figure 1B) and client software (Figure 1C).

#### Image acquisition device

**Figure 2:**
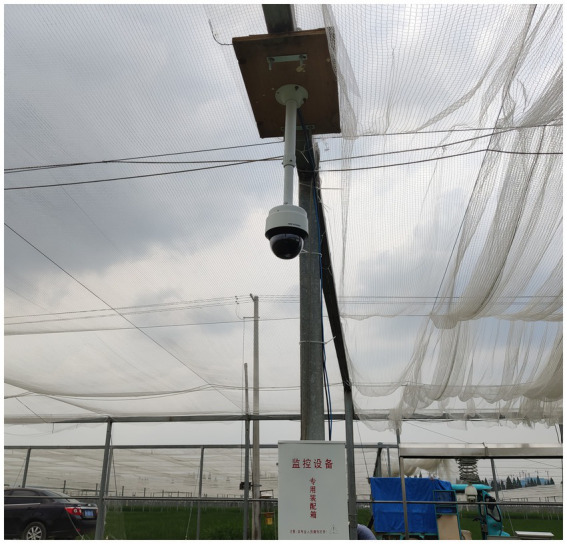
Image acquisition in paddy field.

The image acquisition device is composed of a high-definition network camera (IDS-2DC7823IX-A/T3, HIKVISION, China), a 4G wireless industrial router (R300A, Oray, China) and an equipment box (Figure 2). The network camera was fixed on a pole with a height of 2.7 m in the middle of the paddy field. The 4G wireless industrial router, power supply and other equipment are placed in the equipment box. The camera has 1/1.8″ progressive scan CMOS, 3840 × 2,160 image resolution, 6 mm to 138 mm focal length, 30 frame per second, 23× optical zoom, 300 preset positions and timing snapshots. It supports 360° horizontal rotation and vertical rotation from −5° to −90°. The effective recognition range of rice canopy images captured by each camera is a paddy field of about 310 m^2^, the area of a circle with 20 m diameter. Each uncompressed image is sent to the server and consumes about 3 MB of data traffic.

#### Cloud server

The cloud server is responsible for storing data, controlling devices, running detection models. The system was designed with the front-end and back-end separation architecture mode. The back-end design of the system adopted the Spring + SpringMVC + MyBatis (SSM) architecture. The database used the relational database MySQL.

When the server receives the image acquisition information from the client, the server sends a control signal to the image acquisition device. After the network camera finished the acquisition of rice canopy images, the images are sent to the cloud server. After receiving the images, the server immediately calls the rice diseases and pests detection model and saves the detection results to the database.

#### Client software

The client software was designed based on the Vue framework and JavaScript language for front-end and back-end data interaction and processing. The web page rendering was completed using HTML and CSS. The client-end sent data to the back-end through Axios network request based on HTTP protocol. The data resources include the login interface, detection interface of history and so on. The response data is generated in JSON format and returned to the client-end. After the client-end parses the response data, it renders the response data to the browser interface. The client software realizes the dynamic display of the monitoring results of rice disease and pest lesions. When using this system for the first time, users only need to manually set multiple preset positions with different lens angles and focal lengths in the client software. Then the system can automatically work.

### Image acquisition

Rice canopy images of paddy fields in Hangzhou City, Zhejiang Province, China (38° 53′ 25.06″ N, 119° 56′ 6.25″ E) were collected through our image acquisition device. The size of images is 3,840 × 2,160 pixels. Because rice diseases and pests may appear in the whole rice growth period, we collect the rice canopy images in different weathers and time from 8 a.m. to 6 p.m. every day from May 2021 to October 2021. In order to get more image features of disease and pest lesions, the rice canopy was photographed at multiple scales with the network camera fixed on the pole. We collected over 18,000 rice canopy images.

**Figure 3:**
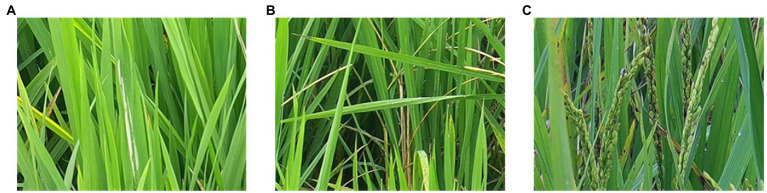
Lesions of three species of diseases and pests on rice canopy. **(A)** Lesions of *Cnaphalocrocis medinalis.* **(B)** Lesions of *Chilo suppressalis.* **(C)** Lesions of *Ustilaginoidea virens.*

In this work, two pest damage lesions (*Cnaphalocrocis medinalis* and *Chilo suppressalis*) and one disease lesion (*Ustilaginoidea virens*) were studied. The three lesions on the rice canopy can be captured by the camera. The lesions of *C. medinalis* present rolled leaves and white lesions on rice leaves (Figure 3A). The lesions of *C. suppressalis* appear dead heart, dead booting, or white panicle of rice plant (Figure 3B). The disease lesions of *U. virens* show yellow or dark green on rice spikes infected by chlamydospores (Figure 3C).

### Data preprocessing

Rice disease and pest lesions in each image were recognized by three technicians at the same time. If they disagreed on the species of one lesion, we adopted the same result recognized by two technicians or abandoned the result. The 4,725 images containing disease or pest lesions among 18,000 images were selected and the lesion regions of each image are annotated by technicians using the LabelImg tool.^1^ Each lesion corresponds to a unique category and a bounding box coordinate. Each image may contain multiple lesions. An annotation file containing bounding boxes and the categories of each lesion was generated for each image. We annotated 22,919 lesions on these images. The lesion number of each disease or pest is listed in Table 1.

**Table 1:** Number of annotated lesions of three diseases and pests.

Disease or pest	*Cnaphalocrocis medinalis*	*Chilo suppressalis*	*Ustilaginoidea virens*
Lesion number	9,739	8,957	4,223

All images were divided into a training set, a validation set and a test set in the ratio of 7:2:1. To overcome the overfitting problem in the training stage of CNNs, data augmentation techniques are often operated on the training set. Because our images were collected in different weather and time, they show different brightness and sharpness. Four augmentation techniques of brightness enhancement, brightness attenuation, contrast enhancement and contrast attenuation (Casado-Garcia et al., 2019) were performed on the training set.

### Detection model for disease and pest lesions of rice canopy

**Figure 4:**
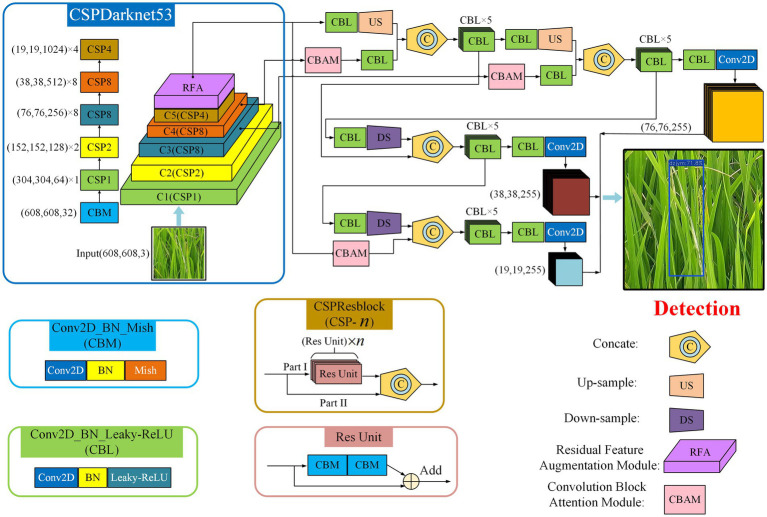
The network structure of YOLO-DPD model.

To achieve the real-time monitoring and high detection accuracy of rice diseases and pests in paddy fields, YOLOv4 (Bochkovskiy et al., 2020) was selected as the basic detection network. However, the shapes of the three lesions are narrow and long. Some lesions have a small proportion in rectangular box annotation and complex surrounding background. The original YOLOv4 shows a low detection accuracy. Many lesions are missed or falsely detected. So we proposed an improved model YOLO-Disease and Pest Detection (YOLO-DPD). The residual feature augmentation method and the attention mechanism were combined to improve the basic detection network of YOLOv4. The detailed design methods of the residual feature augmentation method and attention mechanism are given below. The overall block diagram of the YOLO-DPD detection model of rice disease and pest lesions is shown in Figure 4. The Conv2D_BN_Mish (CBM) block is composed of Conv, BN and Mish activation function. This block uses the Mish activation function to avoid the gradient disappearance in model training. The Conv2D_BN_Leaky_ReLU (CBL) block is composed of Conv, BN and Leaky_Relu activation functions. The model adopts the Leaky_Relu activation function to improve the calculation speed. The Res Unit block enables the network to be built deeper. The CSPResblock (CSP- n ) block consists of CBM components and n the Res Unit blocks.

Firstly, the input image is resized to 608 × 608 pixels, and input into the CSPDarkent53 network to extract features. Secondly, these features are enhanced through the residual feature augmentation and attention mechanism, and the weights of feature channels are assigned according to their importance. Finally, multiple-scale features are fused to obtain a fusion feature map, which is used to predict the location of the disease and pest lesions in the rice canopy image.

#### Residual feature augmentation method

In the deep neural network, features of different layers are extracted from the original images. The lower feature layers have higher resolution and retain more features in the original image, which is helpful to locate the lesions. The higher the feature layer, the smaller the size of the feature map and the richer the semantic information. It is helpful to identify the lesion category, but it is easy to lose the feature information of some small targets. In the original YOLOv4 network structure, the features at the highest level are propagated in a top-down path and gradually fused with the features at lower levels. The features at lower levels also adjust the number of channels by 1 × 1 convolution and down-sampling operation. And then features at lower levels are fused with the features at higher levels. The feature maps at lower levels are enhanced by the semantic information at higher levels, which gives them diverse contextual information. Meanwhile, the target location information of lower layers also enhances that of higher layers. Although the Spatial pyramid pooling (SPP) operation in YOLOv4 greatly increases the receptive field, it also loses detailed information after max pooling (Msonda et al., 2020).

In this paper, the three species of rice disease and pest lesions are mostly small in aspect ratio. The max-pooling operation will lead to the loss of lesion edge information. Before fusing features, if the features of different levels independently perform 1 × 1 convolution to reduce the number of channels, it will result in the loss of some information, the features only contain the context information of a single scale. This information does not consider the huge semantic gap between these features. Directly fusing these features will reduce the ability of multi-scale feature representation, which is not fully compatible with the features at other levels.

**Figure 5:**
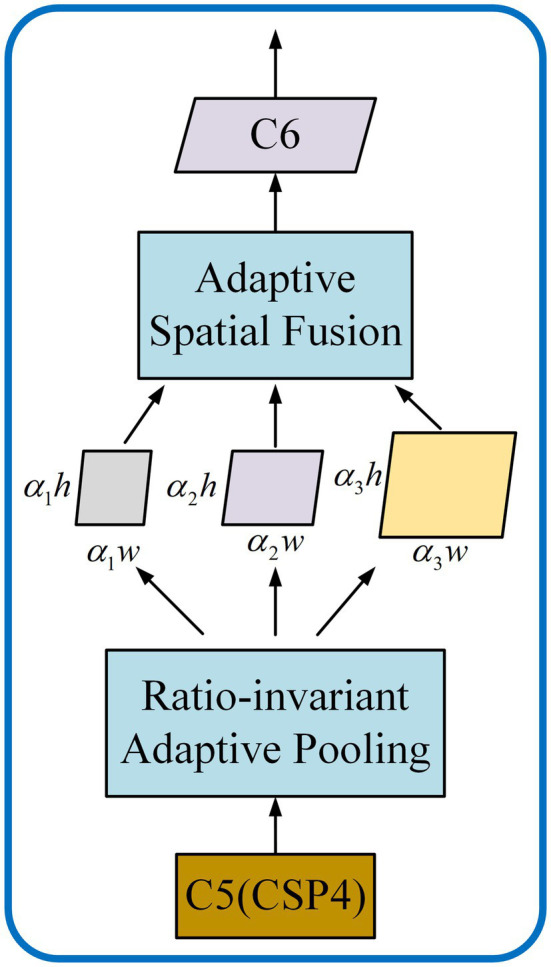
Structure diagram of Residual Feature Augmentation module.

Based on the YOLOv4, the residual feature augmentation (RFA) module (Guo et al., 2020; Figure 5) is used to replace the SPP in the original YOLOv4, and the residual branch is used to inject different spatial context information into the original branch. It improves the feature representation reduces the information loss at a high level and improves the performance of the generated feature pyramid.

**Figure 6:**
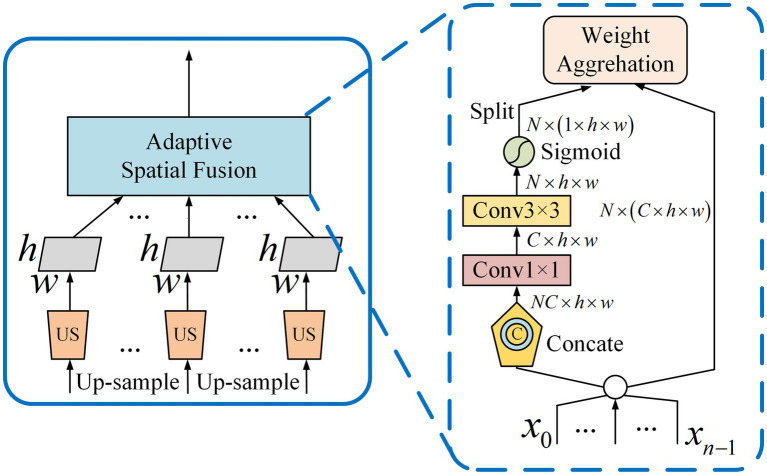
Adaptive Spatial Fusion Module.

In RFA module, multiple context features of different scales are firstly generated by CSPDarknet53. By performing ratio-invariant adaptive pooling on C5 whose scale is *S* (19, 19, 1,024), multiple contexts feature with different scales ( $\alpha_{1}\times S$ , $\alpha_{2}\times S$ , …, $\alpha_{n}\times S$ ) are generated. Then, the number of feature channels is adjusted to 256 by a 1 × 1 convolution. Finally, it is up-sampled to a scale by bilinear interpolation for subsequent fusion. Considering the aliasing effect caused by interpolation, these contextual features are adaptively combined with Adaptive Spatial Fusion (ASF) instead of simple summation (Guo et al., 2020). The detailed structure of the ASF is shown in Figure 6.

ASF takes the up-sampling features as input. First, through the Concat processing, the context features of different scales are stacked. Then the number of channels is adjusted by using a 1 × 1 convolution and the features are further extracted through a 3 × 3 convolution. Finally, the spatial weight maps of each feature are generated through the Sigmoid activation function. The weights aggregate the contextual features into C6 and gave it multi-scale contextual information. After ASF outputs C6, it continues to be fused with other low-level features.

#### Attention mechanism

**Figure 7:**
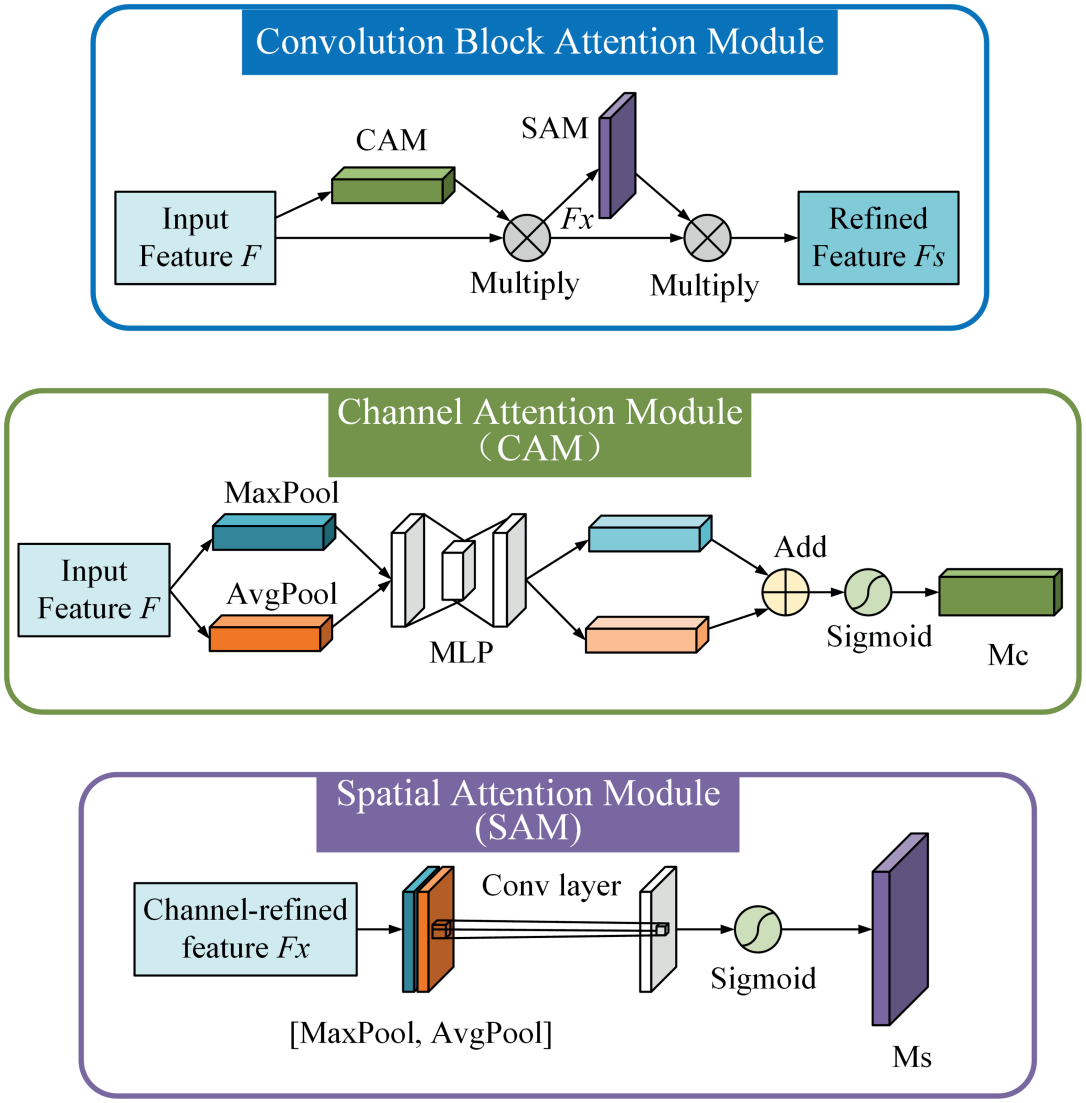
Convolution block attention module.

In the process of multi-scale feature fusion, the feature map obtained by up-sampling and the other feature map extracted by the CSPDarknet53 backbone network is directly channel spliced. It results in a feature map with a large gap in the fusion information of each channel. Different channel fusion features have different importance for different scale detection and identification. In order to make the model pay more attention to the characteristics of the rice disease and pest lesions, a lightweight attention mechanism, convolution block attention module (CBAM; Woo et al., 2018) is added to the feature fusion process (Figure 4). The structure of CBAM is shown in Figure 7.

The CBAM has two sequential sub-modules: a channel attention module (CAM) and a spatial attention module (SAM). The CBAM sequentially infers attention maps along two separate dimensions, channel and spatial. Then the attention maps are multiplied by the input feature map for adaptive feature refinement.

In CAM, the average pooling and max pooling operations are simultaneously used to aggregate channel and spatial information. The average-pooled features and max-pooled features are forwarded to a shared network which was composed of a multi-layer perceptron (MLP). The output feature vectors are merged by element-wise summation and the channel attention map M_c_ is produced.

In SAM, the average pooling and max pooling operations are applied along the channel axis to generate two 2D maps. Then the average-pooled features and max-pooled features are concatenated and convolved by a standard convolution layer. Finally, a spatial attention map M_s_ is generated.

Because CBAM is a lightweight and general module, it can be integrated into any CNN architecture seamlessly with negligible overheads, and it is end-to-end trainable along with base CNNs. We add CBAM after the CSPDarknet53 module in YOLO-DPD to further narrow the semantic gap between different feature layers and improve the saliency of diseases and pests on rice canopy.

### Comparison with different detection model

To compare the detection performance of different models, we fine-tuned the pre-trained RetinaNet (Lin et al., 2020b), Faster R-CNN (Ren et al., 2017), YOLOv4 (Bochkovskiy et al., 2020a), YOLOv4-RFA (The RFA Method was added to YOLOv4) and YOLO-DPD.

### Model training

The experiments were conducted on a deep learning server, which the configuration parameters are shown in Table 2. The software environments include Ubuntu 16.04, python, OpenCV, CUDA, PyTorch, etc. In addition, the PyTorch deep learning framework was used to implement the YOLO-DPD model, which was convenient for the development of comparative experiments due to its Python interfaces.

**Table 2:** Configuration parameters of experimental hardware environment.

Hardware name	Model	Number
Main board	Gigabyte X299-WU8	1
CPU	Intel I7-9800X	1
Memory	Kingston 16G DDR4	4
Graphics card	GeForce GTX1080Ti	4
Solid state drives	Kingston 1 T	1
Hard disk	Western digital 4 T	2

We trained all models with the same hyperparameters. The optimizer was the stochastic gradient descent (SGD) method, the batch size was set to 32, the number of iterations was 1,000, the initial learning rate was 0.001, the gamma was 0.1, the momentum was 0.9 and the weight decay rate was 0.0005.

### Evaluation protocol

To objectively evaluate the detection and identification effect of our YOLO-DPD model, the average precision (*AP*) and the mean average precision (m*AP*) were calculated by the following formulas. The precision (*P*) and recall (*R*) rate, the calculation formulas are such as formulas (1), (2), (3), (4).


(1) $$P=\frac{TP}{TP+FP}$$

(2) $$R=\frac{TP}{TP+FN}$$

(3) $$AP=\int_{0}^{1}P\left(R\right)dR$$

(4) $$\mathrm{mAP}=\frac{\sum_{i=1}^{N}AP_{i}}{N}$$

where *P* denotes the precision and R denotes the recall. *TP* (true positive) represents the number of lesions correctly detected by the model. *FP* (false positive) represents the number of lesions falsely detected by the model, and *FN* (false negative) represents the number of lesions that are not detected. *N* represents the number of lesion species. *mAP* is calculated by the mean of three *AP* values.

## Result

### Model evaluation

#### Detection results of YOLO-DPD

The 472 images of rice canopy disease and pest lesions were tested by our YOLO-DPD model. There are 2,201 lesions on these images, which includes 947 lesions of *C. medinalis*, 851 lesions of *C. suppressalis* and 403 lesions of *U. virens*.

Table 3 shows the precision, recall and average precision of three lesions. We find that YOLO-DPD can achieve good detection results of three lesions, which proves that YOLO-DPD has a strong ability to identify rice diseases and pests from rice canopy images collected in the paddy fields. The detection of *C. medinalis* lesions achieves the highest average precision of 92.24% because *C. medinalis* lesion has white color and often appears on rice leaves. Although the average precision of *C. suppressalis* lesions is the lowest, it is still over 85%, which means most of them detected by our model are correct. The lesions of *C. suppressalis* often appear in the dead heart of rice plants. The symptoms are similar to the withered leaves in the later stage of rice growth, which easily causes false detection.

**Table 3:** Detection results of rice canopy diseases and pests.

Lesion categories	Precision (%)	Recall (%)	Average precision (%)
*Cnaphalocrocis medinalis*	93.05	89.62	92.24
*Chilo suppressalis*	90.61	85.63	87.35
*Ustilaginoidea virens*	91.75	90.04	90.74

**Figure 8:**
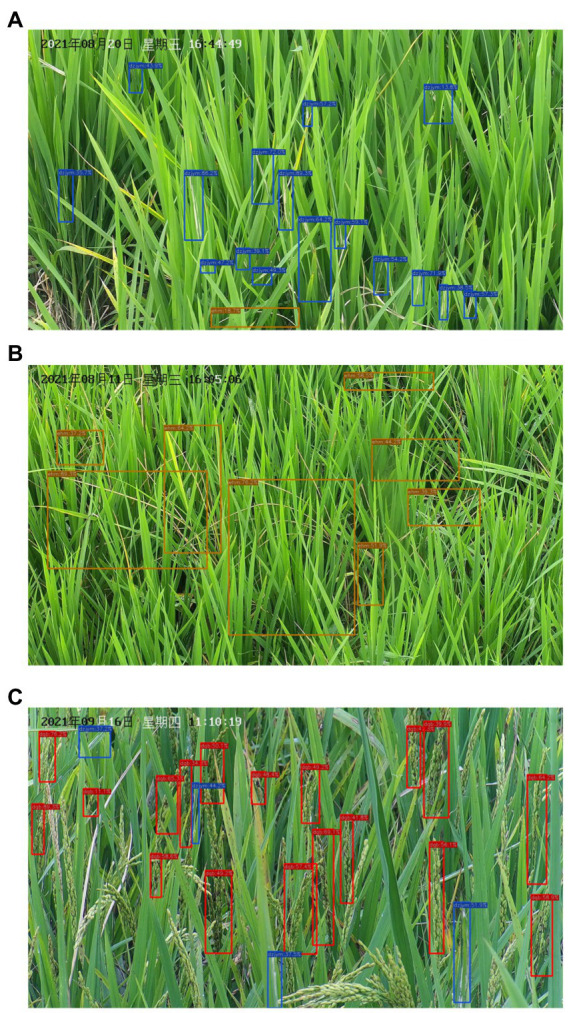
The detection results of three lesions by YOLO-DPD model. Most of **(A)** detected lesions of *C. medinalis*; **(B)** detected lesions of *C. suppressalis*; **(C)** detected lesions of *U. virens.*

Figure 8 gives three examples of three species of lesions detected by YOLO-DPD. The blue, orange, red boxes contain lesions of *C. medinalis*, *C. suppressalis* and *U. virens,* respectively. In Figure 8, we find most of lesions are correctly detected. Two or three species of lesions may appear in one image.

#### Performance comparison of different models

The test results of the five models are shown in Table 4. It can be seen that the YOLO-DPD has higher detection precision than other models for three lesions of diseases and pests on rice canopy. The results prove that YOLO-DPD has the strong generalization ability and robustness. Compared with the original YOLOv4 network, the mAP of YOLO-DPD is increased by 6.1%. So the residual feature augmentation method and attention mechanism of YOLO-DPD improve the detection precision. Compared to RetinaNet, Faster R-CNN and Yolov4 models, the mean average precision of YOLO-DPD increased by 18.20, 6.98, 6.10%, respectively.

**Table 4:** Detection results of 5 different models.

Model	Average precision of different lesions (%)			Average detection time of each image (ms)	Mean average precision mAP (%)
	*Cnaphalocrocis medinalis*	*Chilo suppressalis*	*Ustilaginoidea virens*		
RetinaNet	75.84	68.53	71.36	241	71.91
Faster R-CNN	85.15	79.29	84.95	486	83.13
YOLOv4	84.18	81.85	86.01	39	84.01
YOLOv4 + RFA	90.62	86.39	89.78	43	88.93
YOLO-DPD	92.24	87.35	90.74	47	90.11

The average detection time for one image by YOLO-DPD is only 47 ms, which can meet the real-time requirements and have a good detection effect. From the experimental results, it can be concluded that after adding the residual feature augmentation method and attention mechanism to the original YOLOv4 network, the *mAP* of the three lesions of rice disease and pest on the test set was improved.

### Client software

**Figure 9:**
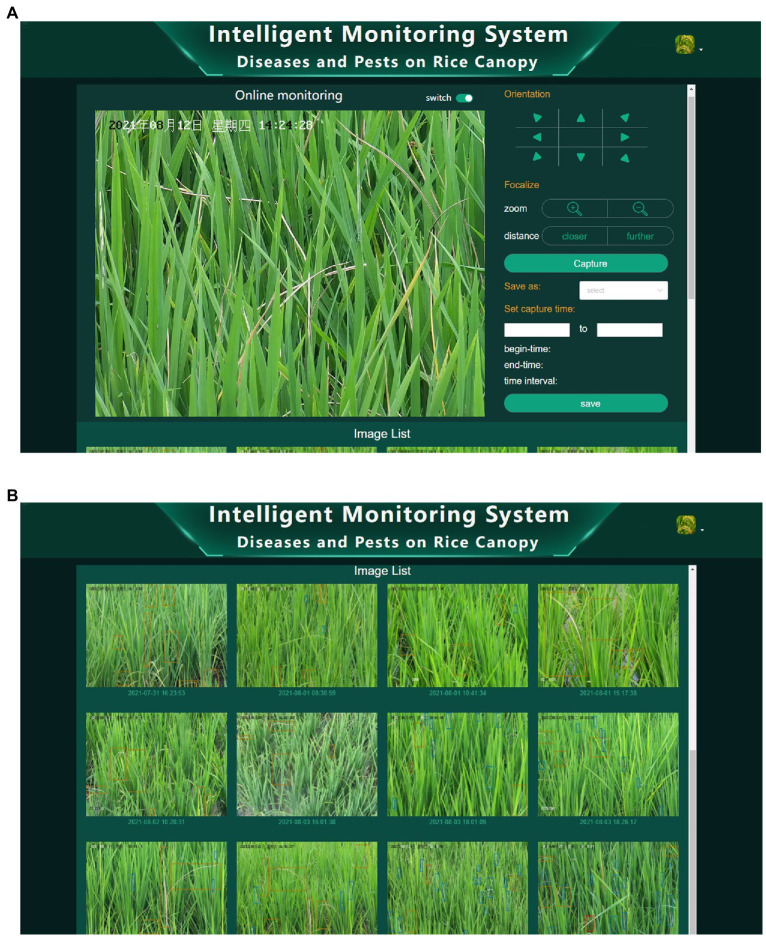
The intelligent monitoring system of diseases and pests on rice canopy. **(A)** Client software interface; **(B)** list of detection results.

To visually present the detection results of YOLO-DPD, a client software was developed for automatically monitoring diseases and pests on the rice canopy. Figure 9A shows the captured rice canopy image by one camera. The multiple positions, zoom sizes and captured time of images can be preset through the client software. The system automatically captures rice canopy images, detects and identifies the disease and pest lesions on images according to the preset parameters. Users can view the detection results on the rice canopy images in real-time through the Web page, which realizes the traceability of data. Figure 9B shows the detected image list.

### Comparison of different monitoring methods

To verify the advantages of our system, we compared some characteristics of three different monitoring methods including manual, computer vision-based and our methods in Table 5. Compared with the other two monitoring methods, our method does not require any manual operations during the monitoring of rice diseases and pests. It takes the shortest time to monitor and identify the disease and pest lesions in the same area of paddy fields. The monitoring process is not affected by time and weather. And it will not disturbance with rice’s normal growth. Users can easily trace historical data on the client. In conclusion, our system has the remarkable characteristics of automatic photography at multiple locations and times, high efficiency, labor-saving and non-destructive monitoring of diseases and pests on the rice canopy.

**Table 5:** Comparison of different monitoring methods.

Characteristics	Different monitoring methods		
	Manual survey	Computer vision-based	Our
Image acquisition tool	No	Camera, mobile phone, UAV	Network camera
Number of workers	2	1	0
Time consumption	About 5 min/m^2^	Uncertainty	0.5 min/m^2^
Coverage area	Any area	Any area	310m^2^ / per camera
Precision	High	High	High
Subjectivity	High	No	No
Monitoring time	Restricted	Restricted	Unrestricted
Weather influence	In thunder and rain weathers, surveys are dangerous	In thunder and rain weathers, surveys are dangerous	No
Disturbance to rice growth	Big	Camera and mobile phone: Big UAV: Medium	No
Data format	Manually recording data	Images	Images
Traceability of historical data	Hard	Easy	Easy
Reference	http://openstd.samr.gov.cn/bzgk/gb/newGbInfo?hcno=02665E5D6CFAD6BC44DAE48435461132; http://openstd.samr.gov.cn/bzgk/gb/newGbInfo?hcno=DECC23CBB9712996CF725DBBD67A974E; http://bzxx.ahbz.org.cn/searchListDetail.html?type=0&id=11825995	Dong et al. (2020); Bhoi et al. (2021); Chen et al. (2021); Daniya and Vigneshwari, (2021); Wang et al. (2021)	/

## Conclusion and future work

This study aims to solve the time-consuming, laborious and subjective problems caused by the manual field survey of three lesions on the rice canopy. We developed an intelligent monitoring system for monitoring the diseases and pests on the rice canopy. The system can automatically collect rice canopy images at the preset multiple locations and times. To accurately detect and identify three lesions of diseases and pests on the rice canopy, we proposed a YOLO-DPD model with the residual feature augmentation method and attention mechanism module based on the YOLOv4 model. The improved YOLO-DPD makes the network pay more attention to the features of rice disease and pest lesions. The results show that YOLO-DPD can obtain the mean average precision of 90.11% on the test set and adapt to the complex paddy fields. The users can view the detection results on the client software interface.

The system reduces the workload of technicians and saves the disease and pest images in paddy fields. The system provides reliable data for early warning and real-time effective control of rice diseases and pests.

Although some progress of disease and pest lesion detection on the rice canopy had been made in this paper, we need to furtherly study the accuracy detection of occluded lesions, similar symptoms of healthy leaves and very small lesions. Some occluded lesions in the rice tillering stage may cause some missing detection. Normal physiological yellow leaves may cause some error detection. We will collect more images for improving the detection precision. The warning model of diseases and pests on the rice canopy should be developed according to our detection results for deciding the controlling measures of rice diseases and pests. In the future, we will focus on solving these problems for automatically and accurately monitoring rice diseases and pests.

## Data availability statement

The raw data supporting the conclusions of this article will be made available by the authors, without undue reservation.

## Author contributions

SL, HL, and QY proposed the detection model and wrote and revised the manuscript. BY, SL, and JT contributed to paddy fields surveys and manual data annotation. ZF, FL, and YG developed the system software. All authors contributed to the article and approved the submitted version.

## Funding

This study is supported by the Natural Science Foundation of Zhejiang, China (No. LY20C140008), the National Key Research Program of China during the 14th Five-Year Plan Period (No. 2021YFD1401100), and the Industry-Academia-Research Cooperation Project of Zhuhai, China (No. ZH22017001210013PWC).

## Conflict of interest

The authors declare that the research was conducted in the absence of any commercial or financial relationships that could be construed as a potential conflict of interest.

## Publisher’s note

All claims expressed in this article are solely those of the authors and do not necessarily represent those of their affiliated organizations, or those of the publisher, the editors and the reviewers. Any product that may be evaluated in this article, or claim that may be made by its manufacturer, is not guaranteed or endorsed by the publisher.

## Footnotes

^1^ https://github.com/tzutalin/labelImg

## References

[ref1] Abd El-Ghany N. M. Abd El-Aziz S. E. Marei S. S. (2020). A review: application of remote sensing as a promising strategy for insect pests and diseases management. Environ. Sci. Pollut. Res. 27, 33503–33515. doi: 10.1007/s11356-020-09517-2, PMID: 32564316

[ref2] Aboutalebi M. Torres-Rua A. E. McKee M. Kustas W. P. Nieto H. Alsina M. M.. (2020). Incorporation of unmanned aerial vehicle (UAV) point cloud products into remote sensing evapotranspiration models. Remote Sens. 12:50. doi: 10.3390/rs12010050, PMID: 32355570 PMC7192004

[ref3] Ali M. M. Bachik N. A. Muhadi N. A. Yusof T. N. T. Gomes C. (2019). Non-destructive techniques of detecting plant diseases: A review. Physiol. Mol. Plant Pathol. 108, 101426. doi: 10.1016/j.pmpp.2019.101426

[ref4] Bari B. S. Islam M. N. Rashid M. Hasan M. J. Razman M. A. M. Musa R. M.. (2021). A real-time approach of diagnosing rice leaf disease using deep learning-based faster R-CNN framework. PeerJ Comput. Sci. 7, e432. doi: 10.7717/peerj-cs.432, PMID: 33954231 PMC8049121

[ref5] Bhoi S. K. Jena K. K. Panda S. K. Long H. V. Kumar R. Subbulakshmi P.. (2021). An internet of things assisted unmanned aerial vehicle based artificial intelligence model for rice pest detection. Microprocess. Microsyst. 80, 103607. doi: 10.1016/j.micpro.2020.103607

[ref6] Bochkovskiy A. Wang C. -Y. Liao H. -Y. M. (2020). Yolov4: Optimal speed and accuracy of object detection. *arXiv* [Epub ahead of print]. doi: 10.48550/arXiv.2004.10934

[ref7] Casado-Garcia A. Dominguez C. Garcia-Dominguez M. Heras J. Ines A. Mata E.. (2019). CLoDSA: a tool for augmentation in classification, localization, detection, semantic segmentation and instance segmentation tasks. BMC Bioinform. 20, 323. doi: 10.1186/s12859-019-2931-1, PMID: 31195959 PMC6567576

[ref8] Chen J. W. Lin W. J. Cheng H. J. Hung C. L. Lin C. Y. Chen S. P. (2021). A smartphone-based application for scale Pest detection using multiple-object detection methods. Electronics 10:372. doi: 10.3390/electronics10040372

[ref9] Daniya T. Vigneshwari S. (2021). Deep neural network for disease detection in Rice Plant using the texture and deep features. Comput. J. 65, 1812–1825. doi: 10.1093/comjnl/bxab022

[ref10] Dong Y. Y. Xu F. Liu L. Y. Du X. P. Ren B. Y. Guo A. T.. (2020). Automatic system for crop Pest and disease dynamic monitoring and early forecasting. IEEE 13, 4410–4418. doi: 10.1109/JSTARS.2020.3013340

[ref11] Gao D. M. Sun Q. Hu B. Zhang S. (2020). A framework for agricultural Pest and disease monitoring based on internet-of-things and unmanned aerial vehicles. Sensors 20:1487. doi: 10.3390/s20051487, PMID: 32182732 PMC7085563

[ref12] Guo C. Fan B. Zhang Q. Xiang S. Pan C. (2020). "Augfpn: Improving multi-scale feature learning for object detection", in: *Proceedings of the IEEE/CVF Conference on Computer Vision and Pattern Recognition*, 12595–12604.

[ref13] Huang Y. B. Reddy K. N. Fletcher R. S. Pennington D. (2018). UAV low-altitude remote sensing for precision Weed Management. Weed Technol. 32, 2–6. doi: 10.1017/wet.2017.89

[ref14] Jiang Y. Liu W. Huang C. Lu M. Liu J. Ci R. (2020). Forecast of occurrence trend of major crop diseases and pests in 2020. China Plant Protect. 40, 37–39+53. doi: 10.3969/j.issn.1672-6820.2020.02.007

[ref15] Joshi R. C. Kaushik M. Dutta M. K. Srivastava A. Choudhary N. (2021). VirLeafNet: automatic analysis and viral disease diagnosis using deep-learning in Vigna mungo plant. Ecol. Inform. 61, 101197. doi: 10.1016/j.ecoinf.2020.101197

[ref16] Khaki S. Pham H. Han Y. Kuhl A. Kent W. Wang L. Z. (2021). DeepCorn: A semi-supervised deep learning method for high-throughput image-based corn kernel counting and yield estimation. Knowl.-Based Syst. 218, 106874. doi: 10.1016/j.knosys.2021.106874

[ref17] Li D. S. Wang R. J. Xie C. J. Liu L. Zhang J. Li R.. (2020). A recognition method for Rice Plant diseases and pests video detection based on deep convolutional neural network. Sensors 20:578. doi: 10.3390/s20030578, PMID: 31973039 PMC7038217

[ref18] Lin T.-Y. Goyal P. Girshick R. He K. Dollar P. (2020b). Focal loss for dense object detection. IEEE Trans. Pattern Anal. Mach. Intell. 42, 318–327. doi: 10.1109/tpami.2018.2858826, PMID: 30040631

[ref19] Lin F. F. Guo S. Tan C. W. Zhou X. G. Zhang D. Y. (2020a). Identification of Rice sheath blight through spectral responses using hyperspectral images. Sensors 20:243. doi: 10.3390/s20216243, PMID: 33147714 PMC7663646

[ref20] Liu W. Lu M. Huang C. Yang Q. (2020). Construction and application of cross border and cross regional monitoring and early warning system for major rice diseases and pests. Plant Prot. 46, 87–92+100. doi: 10.16688/j.zwbh.2019457

[ref21] Liu J. Wang X. W. (2020a). Early recognition of tomato gray leaf spot disease based on MobileNetv2-YOLOv3 model. Plant Methods 16, 83. doi: 10.1186/s13007-020-00624-2, PMID: 32523613 PMC7281931

[ref22] Liu J. Wang X. W. (2020b). Tomato diseases and pests detection based on improved Yolo V3 convolutional neural network. Front. Plant Sci. 11:898. doi: 10.3389/fpls.2020.00898, PMID: 32612632 PMC7309963

[ref23] Lu Y. Yi S. J. Zeng N. Y. Liu Y. R. Zhang Y. (2017). Identification of rice diseases using deep convolutional neural networks. Neurocomputing 267, 378–384. doi: 10.1016/j.neucom.2017.06.023, PMID: 35356111

[ref24] Morsy M. (2020). Monitoring and managing Rice Pest infestation through hyperspectral remote sensing technology under field conditions. J. Appl. Plant Protect. 9, 67–82. doi: 10.21608/japp.2020.178429

[ref25] Msonda P. Uymaz S. A. Karaagac S. S. (2020). Spatial pyramid pooling in deep convolutional networks for automatic tuberculosis diagnosis. Traitement Du Signal 37, 1075–1084. doi: 10.18280/ts.370620

[ref26] Ren S. He K. Girshick R. Sun J. (2017). Faster R-CNN: towards real-time object detection with region proposal networks. IEEE Trans. Pattern Anal. Mach. Intell. 39, 1137–1149. doi: 10.1109/TPAMI.2016.2577031, PMID: 27295650

[ref27] Sethy P. K. Barpanda N. K. Rath A. K. Behera S. K. (2020). Image processing techniques for diagnosing Rice Plant disease: A survey. Proc. Comput. Sci. 167, 516–530. doi: 10.1016/j.procs.2020.03.308

[ref28] Tian Y. N. Yang G. D. Wang Z. Li E. Liang Z. Z. (2019). Detection of apple lesions in orchards based on deep learning methods of CycleGAN and YOLOV3-dense. J. Sens. 2019:926. doi: 10.1155/2019/7630926

[ref29] Wang F. Y. Wang R. J. Xie C. J. Zhang J. Li R. Liu L. (2021). Convolutional neural network based automatic pest monitoring system using hand-held mobile image analysis towards non-site-specific wild environment. Comput. Electron. Agric. 187:268. doi: 10.1016/j.compag.2021.106268

[ref30] Woo S. H. Park J. Lee J. Y. Kweon I. S. (2018). CBAM: convolutional block attention module. *15th European Conference on Computer Vision (ECCV)*, 3–19.

[ref31] Yang Q. Shi L. S. Han J. Y. Zha Y. Y. Zhu P. H. (2019). Deep convolutional neural networks for rice grain yield estimation at the ripening stage using UAV-based remotely sensed images. Field Crop Res. 235, 142–153. doi: 10.1016/j.fcr.2019.02.022

[ref32] Yasrab R. Zhang J. C. Smyth P. Pound M. P. (2021). Predicting plant growth from time-series data using deep learning. Remote Sens. 13:331. doi: 10.3390/rs13030331

[ref33] Zhang J. C. Huang Y. B. Pu R. L. Gonzalez-Moreno P. Yuan L. Wu K. H.. (2019). Monitoring plant diseases and pests through remote sensing technology: A review. Comput. Electron. Agric. 165, 104943. doi: 10.1016/j.compag.2019.104943, PMID: 32564316

[ref34] Zhang D. Y. Zhou X. G. Zhang J. Lan Y. B. Xu C. Liang D. (2018). Detection of rice sheath blight using an unmanned aerial system with high-resolution color and multispectral imaging. PLoS One 13, e0187470. doi: 10.1371/journal.pone.0187470, PMID: 29746473 PMC5945033

[ref35] Zhou G. X. Zhang W. Z. Chen A. B. He M. F. Ma X. S. (2019). Rapid detection of Rice disease based on FCM-KM and faster R-CNN fusion. IEEE 7, 143190–143206. doi: 10.1109/ACCESS.2019.2943454

